# Does enhanced HIV prevention, diagnosis, and linkage to care reduce hospitalisation in high HIV-burden communities in Zambia and South Africa? findings from the HPTN 071 (PopART) randomised trial

**DOI:** 10.1371/journal.pgph.0004373

**Published:** 2025-05-08

**Authors:** Ronelle Burger, Nomtha Bell-Mandla, Abigail Harper, Sean Richardson, Sarah Kanema, Ranjeeta Thomas, Lawrence Mwenge, Ethan Wilson, Sian Floyd, Peter Bock, Helen Ayles, Sarah Fidler, Richard Hayes, Katharina Hauck, the HPTN 071 (PopART) study team

**Affiliations:** 1 Economics Department, Stellenbosch University, Stellenbosch, South Africa; 2 Desmond Tutu TB Centre, Department of Paediatrics and Child Health, Stellenbosch University, Cape Town, South Africa; 3 Erasmus School of Health Policy & Management, Erasmus University Rotterdam, Rotterdam, The Netherlands; 4 University of Zambia, Lusaka, Zambia; 5 Department of Health Policy, London School of Economics and Political Science, London, United Kingdom; 6 University Teaching Hospitals, Lusaka, Zambia; 7 Fred Hutchinson Cancer Research Center, Seattle, Washington, United States of America; 8 Department of Infectious Disease Epidemiology, London School of Hygiene and Tropical Medicine, London, United Kingdom; 9 Department of Clinical Research, London School of Hygiene and Tropical Medicine, London, United Kingdom; 10 Zambia AIDS Related Tuberculosis Project, University of Zambia, Lusaka, Zambia; 11 Department of Medicine, Faculty of Medicine, Imperial College, London, United Kingdom; 12 MRC Centre for Global Infectious Disease Analysis & Jameel Institute for Disease and Emergency Analytics, School of Public Health, Imperial College London, London, United Kingdom; University of Cape Town Faculty of Health Sciences, SOUTH AFRICA

## Abstract

The objective of this study is to explore if a community-based HIV combination prevention intervention reduced inpatient hospitalisations in Zambia and South Africa by diagnosing HIV and TB in earlier stages of disease progression, thereby preventing severe disease and new infections. As part of the HIV Prevention Trials Network (HPTN) 071 trial, hospitalisation data from a cohort of 16 968 consenting randomly sampled adults aged 18–44 years were collected between 28 November 2013 and 16 November 2018 across 21 communities in Zambia and South Africa across three study arms. Arm A included annual visits by Community HIV-care Providers (CHiPs) and universal linkage to care for ART initiation for all PLWH (irrespective of CD4 count); arm B included annual CHiPs visits and ART per local guidelines; control arm C received the standard of care provided at government clinics, including HIV testing and ART offered according to local guidelines. For this study, we used a cluster-level two-stage analysis and adjusted for covariates that were unbalanced across intervention arms. Covariates included in the models were the cluster’s baseline HIV prevalence and hospitalisation rate and data on the respondent’s gender, age, educational attainment, and socio-economic status. Out of the pooled sample of 13 964 responses from the three post-baseline surveys, 439 (3.14%) reported hospitalisation in the past 12 months – 234 (1.68%) when excluding hospital admissions for births or injury. Comparing hospitalisations in the intervention and control arm clusters, the estimated adjusted risk ratio was 1.03 [0.64–1.66] for the full sample and 0.82 [0.39–1.74] for PLWH. We find no compelling evidence of impact of the HPTN071 (PopART) community-wide combination HIV prevention intervention on in-patient hospitalisation among a general population sample.

**Trial registration:**

ClinicalTrials.gov NCT01900977

## Introduction

There has been significant progress in the management of HIV and access to antiretroviral therapy (ART) globally over the past decade. [1,2,3]. People living with HIV (PLWH) are living longer and with a better quality of life due to advances in treatment and much-improved access to ART. Without ART most PLWH progress to AIDS within the first decade from diagnosis and typically die within 2 years of an AIDS diagnosis [4]. A systematic review of the virological efficacy of ART found that 78% of patients achieved virological suppression after 6 months of antiretroviral therapy [5]. ART enables PLWH to achieve the same average life expectancy as people who do not have HIV [4,6]: A 35-year-old HIV-positive person who is successfully treated - achieving viral suppression and a CD4 + cell count of at least 350 cells/μL a year after initiating ART - has a life expectancy of 80 [7].

Despite advances in ART access and a sharp decline in AIDS-related deaths, HIV continues to rank amongst the leading causes of death in African countries [8]. Large gaps remain in the prevention and management of HIV and in access to care, especially in high-burden regions such as eastern and southern Africa, home to more than half of the world’s PLWH population. Consequently, many PLWH are diagnosed late, do not initiate treatment, and do not achieve viral suppression [9]. In 2023, 93% [75–98%] of PLWH in this region knew their HIV status, but only 83% [68–96%] of those who knew their status were accessing treatment and 78% [72–86%] of PLWH accessing treatment were virally suppressed [10].

The objective of this study was to assess whether the HPTN 071 (PopART) trial’s combination HIV intervention affected hospitalisations in the study communities in South Africa and Zambia. At the time of the baseline survey, in 2013, South Africa had an adult HIV prevalence of 19% [18%–20%] with an estimated 40% of PLWH on treatment. In Zambia the adult HIV prevalence was 13% [12%–14%] in 2013, with half of PLWH on treatment [11].

The intervention arms included annual home-based HIV testing and TB screening and linkage to care. Community health workers followed up with HIV-positive individuals to support linkage to HIV care and retention on ART. The intervention also promoted services for the prevention of mother-to-child transmission (PMTCT) to pregnant women with HIV and voluntary medical male circumcision for HIV-negative men. Condoms were provided and individuals with sexually transmitted infections (STIs) were referred to health facilities for treatment. Intervention Arm A offered universal ART and intervention arm B offered ART according to national guidelines. The interventions had a significant impact on treatment coverage and viral suppression in both intervention arms. Compared to the arm C communities who received the standard of care, the combined Arms A and B showed an overall reduction of 20% in HIV incidence [12].

This study adds to the broader literature assessing the health system benefits of HIV prevention and earlier HIV detection in low and -middle income countries with limited resources and a high disease burden. Hospitalisation rates are higher for PLWH who initiate ART later compared with those initiating earlier [9,13,14]. Given the potential financial impact of earlier access to care and disease prevention in resource-poor health systems, such evidence is urgently needed.

We identified three hypothetical mechanisms hrough which the Community HIV-care Providers (CHiPs) intervention to have an impact on reduced hospitalisation: (a) by preventing advanced HIV disease and related opportunistic infections through earlier diagnosis, ART initiation, and improvedadherence, (b) by preventing new HIV infection in HIV-negative individuals; and (c) by enabling earlier diagnosis and treatment of TB in both PLHIV and HIV-negative individuals. However, given that TB prevalence was low in the study population (1% of respondents reported a TB diagnosis at baselinecompared to 13% testing positive for HIV), this mechanisms is expected to have a small impact of hospitalisations. Similarly, reductions in HIV incidence are unlikely to translate into fewer hospitalisations within the 3-year time window of this study given that HIV disease progresses over several years: without treatment, the average life expectancy of PLWH is approximately 10 years [4,6] and it may take 10 years or more for AIDS to develop after seroconversion [15].

Based on this evidence the most plausible mechanism would be through improved linkage to care of advanced HIV disease cases who have defaulted from care or who have never been in care. Linkage to care for STI cases was not included as a pathway because STIs rarely lead to hospitalisation.

## Methods

The HPTN 071 (PopART) study presented a unique opportunity to assess the impact of HIV early diagnosis and treatment initiation on inpatient hospitalisation in lower- and middle-income countries with a high disease burden. The study, conducted from 28 November 2013–16 November 2018, was a three-arm, cluster-randomized controlled trial evaluating the impact of a Universal Testing and Treatment (UTT) strategy. Two of the study’s intervention arms included a community-based intervention, where CHiPs teams went door-to-door annually to test community members for HIV and screen them for TB and sexually transmitted infections (STIs). HIV-positive individuals were actively followed up to promote treatment initiation and adherence and those who screened positive for TB and STIs were referred to the health facility for diagnosis and treatment. In addition, as part of HIV prevention, condom use was encouraged, condoms were provided and uncircumcised HIV-negative men were referred for voluntary medical male circumcision at the nearest primary health care facility.

The study selected 21 communities, 12 in Zambia and 9 in South Africa to be included in this trial. Together these communities included an estimated resident population of 1 million people. The selection criteria for communities included high HIV prevalence, a health facility that offered services for both TB and HIV and reaching a catchment population of 20 000 or more. An effort was made to select communities that expressed willingness to be involved in this study, were geographically distinct, and were not the subject of other planned or ongoing HIV prevention studies. Communities in each of the countries were matched into triplets based on estimated HIV prevalence and geographic proximity. The trial randomly assigned the 21 urban and peri-urban communities in Zambia and South Africa to one of three groups: Arm A, which included annual CHiPs visits and ART for all PLWH irrespective of CD4 count and in advance of changes to national guidelines; Arm B, which included annual CHiPs visits and ART provided according to local guidelines; and the control group, Arm C, which provided the local standard of care. At the start of the study local treatment guidelines in Zambia and SA restricted ART to PLWH who had advanced HIV disease or immune suppression (a CD4 count below 350 cells/mm³ at the start of the trial and later, 500 cells/mm³) ART for all PLWH was gradually introduced as the local standard of care starting in April 2016 in Zambia and in October 2016 in South Africa.

The primary and secondary endpoints were measured in a randomly selected cohort of approximately 2000 individually consenting adults aged 18–44 years resident within the participating communities at the time of enrolment per community called the population cohort (PC). PC participants were visited 4 times over 3–4 years. At each visit a survey was administered, blood was collected for a fourth-generation laboratory-based HIV test and an on-the-spot HIV rapid test was offered. Fourth-generation HIV tests were conducted at central laboratories in South Africa and Zambia. For quality control, further HIV testing was conducted at the HPTN Laboratory Center in Baltimore using prespecified testing algorithms. These additional tests were also used to confirm incident HIV infections (a change in HIV status from HIV-negative to HIV-positive). More details on laboratory testing protocols are provided elsewhere [16].

The study aimed to retain 90% of participants by clearly explaining the study’s purpose, collecting locator information, and maintaining regular communication to raise awareness about HIV prevention. Retention strategies also included context-specific strategies based on advice from community leaders, including SMS reminders and engagement of household members to support adherence to study visits.

The survey included questions on hospitalisation that were administered to a separate randomly selected 1-in-5 subsample of participants at each PC survey. The pooled data set represents four cross-sectional surveys, administered about 12 months apart (S1 Fig provides more detail on the timing of each of the surveys).

The survey asked individuals “In the last 12 months, how many times were you admitted to hospital or other types of inpatient care and stayed one or more nights?”. Our primary outcome variable is the resulting binary variable, which is 1 if the respondent had stayed in the hospital in the last 12 months for at least one night, a zero if not. The survey also asked the reason for the most recent hospitalisation, and excluded responses where the reason for admission was reported as injury, accident, or giving birth (analysis for hospitalisation including these cases reported in S3 Table).

The units of analysis in this paper were individual responses to the hospitalisation module. The estimation sample for the cluster-level analysis excludes all survey responses that do not include a response to this module and also excludes responses at the baseline survey (PC0). The full PC sample, without these exclusions, includes 162 945 survey responses from 48 301 individuals, with 54 920 responses in Arm A, 54 542 in Arm B, and 53 483 in Arm C (control). Out of these 48 301 individuals, 27 915 were never surveyed about recent hospitalisation and 5 515 were only surveyed at PC0, leaving 14 871 individuals who received one or more hospitalisation modules across PC12, PC24, and PC36. Of these 14 871 individuals, 2 714 (18.25%) never responded to the hospitalisation questions, 10 450 (70.27%) responded once, 1 607 (10.81%) responding twice and 100 (0.67%) responded three times, resulting in an overall estimation sample size of 13 964.

The estimation sample consists of 4 240 responses in Arm A including 856 from PLWH (based on confirmed laboratory results), 4 894 responses in Arm B including 1 095 from PLWH, and 4 830 responses in Arm C including 1 028 PLWH. Out of these responses, there were 134, 139, and 166 reported hospitalisations in Arm A, B, and C, respectively. If we exclude admissions for hospital delivery and injuries, the numbers decrease to 73, 77, and 84 hospitalisations in Arms A, B, and C, respectively. For PLWH, there were 35, 38, and 37 hospitalisations in the respective arms, dropping to 27, 26, and 22 when admissions for hospital delivery and injury were excluded.

To identify the impact of the PopART intervention on inpatient hospitalisation, we assess whether there is a significant difference in the risk of hospitalisation between the intervention and control arm clusters. We estimate risk ratios using a cluster-level two-stage analysis recommended for cluster-randomized trials with fewer than 15 clusters per treatment arm [17]. With this method, we, first, calculate the observed risk of hospitalisation in each cluster by dividing the number of reported hospitalisations by the number of responses to the hospitalisation module. We, secondly, calculate the expected risk of hospitalisation in each cluster under the null hypothesis of no intervention effect; this is done by fitting a logistic regression model that describes the probability of hospitalisation as a function of the respondent’s specific covariate mix (excluding the study arm), and then aggregating the predicted probabilities across all participants in the cluster. We obtain a cluster ratio-residual by dividing the observed hospitalisation risk by the expected risk, with an adjusted risk ratio for each triplet obtained by dividing the ratio-residuals in the intervention arms within the triplet by those of the control arms. The mean of the logarithm of the adjusted triplet risk ratio is the log intervention effect, with a point estimate intervention effect calculated by taking the geometric mean of the adjusted risk ratios. Two-way ANOVA is conducted on the log ratio-residuals to obtain the standard error of the intervention effect, whilst confidence intervals are calculated using a t-distribution, with the logarithm of the upper (lower) bound calculated by adding (subtracting) the product of the chosen t-statistic and the estimated standard error of the logarithm of the risk ratio to (from) the logarithm of the risk ratio.

The dataset used for analysis is a pooled version of the three follow-up PC surveys (PC12-PC36). The baseline survey is not included because we are interested in post-intervention differences. Baseline values for HIV prevalence and hospitalisation were included as covariates at stage 1 to adjust for any imbalance in covariates. To check for year-specific or duration-dependent effects we also estimated models for PC12, PC24, and PC36 separately. All models were estimated both for the full samples and for the HIV-positive subsample. We adjusted for covariates that were both unbalanced across study arms and were expected to have a strong correlation with hospitalisation based on past studies in this literature. The covariates included in the models were the cluster’s baseline HIV prevalence and rate of hospitalisation and individual gender, age, educational attainment, socio-economic status, and triplet categories. We examine hospitalisations in the full sample of survey responses and in the subsample of responses from individuals confirmed to have HIV. We compare outcomes for arms A and C, arms B and C and the combination of arms A and B against arm C. We view the latter comparison as the primary analysis.

HIV prevalence relied on lab-confirmed HIV diagnosis, including confirmatory testing. TB screening and TB diagnosis were both self-reported, based on survey questions asking the respondent whether over the past 12 months, they had been asked about TB symptoms and they had been told that they had TB.

To capture socio-economic status, we use the wealth index (S2 describes the estimation of the index based on a range of variables). We captured educational attainment as a categorical variable: incomplete primary schooling, completed primary schooling, completed secondary schooling, and tertiary education.

The clustering induced by the study design has been adjusted for confidence intervals. Furthermore, logit transformations have been used to account for the binary nature of the outcome variable.

### Ethical considerations

The HPTN 071 (PopART) study was approved by the Stellenbosch University Health Research Ethics Committees (N12/11/074), the London School of Hygiene and Tropical Medicine (6326) ethics committee, the Division of AIDS (DAIDS) (Protocol ID 11865) and the University of Zambia Bioethics committee (reference number 011-11-12). PopART was registered with ClinicalTrials.gov (registration number NCT01900977). All participants included in the PopART population cohort provided written informed consent.

## Results

Out of the pooled sample of 13 964 hospitalisation module responses from the three post-baseline surveys, 439 (3.14%) indicated hospitalisation in the past 12 months. When we exclude admissions for hospital delivery and injury, the number of hospitalisations drops to 234 (1.68%). Table 1 below shows the hospitalisations per survey round and study arm.

**Table 1:** Proportion of hospitalisations per survey and study arm.

		Any hospitalisation over the past 12 months			Any hospitalisation over the past 12 months excluding admissions for hospital delivery, injuries, and accidents			Hospitalisation Module Responses (N)
		Proportion	Lower bound 95% CI	Upper bound 95% CI	Proportion	Lower bound 95% CI	Upper bound 95% CI	
**Full sample: all survey rounds and study arms**		0.0359	0.0295	0.0436	0.0180	0.0150	0.0215	20439
**Baseline (PC0)**	Arm A	0.0442	0.0235	0.0817	0.0231	0.0156	0.0341	1989
	Arm B	0.0380	0.0267	0.0538	0.0156	0.0074	0.0328	2237
	Arm C	0.0542	0.0330	0.0878	0.0231	0.0157	0.0339	2249
**Survey 2 (PC12)** 12 months after baseline	Arm A	0.0344	0.0238	0.0493	0.0179	0.0111	0.0289	1339
	Arm B	0.0342	0.0219	0.0530	0.0194	0.0095	0.0391	1756
	Arm C	0.0341	0.0157	0.0725	0.0148	0.0078	0.0281	1552
**Survey 3 (PC24)** 24 months after baseline	Arm A	0.0314	0.0201	0.0488	0.0192	0.0120	0.0306	1560
	Arm B	0.0292	0.0173	0.0491	0.0162	0.0027	0.0242	1607
	Arm C	0.0375	0.0209	0.0664	0.0196	0.0121	0.0316	1733
**Endline (PC36)** 36 months after baseline	Arm A	0.0291	0.0152	0.0548	0.0142	0.0071	0.0281	1341
	Arm B	0.0209	0.0125	0.0348	0.0111	0.0052	0.0236	1531
	Arm C	0.0311	0.0222	0.0433	0.0175	0.0078	0.0386	1545

Notes: Proportions reported here are overall proportions in each arm. CIs were adjusted for clustering associated with the study design. The analysis sample differs from the full sample because it excludes the baseline. The survey asked individuals “In the last 12 months, how many times were you admitted to hospital or other types of inpatient care and stayed one or more nights?”. Our primary outcome variable is the resulting binary variable, which is 1 if the respondent had stayed in the hospital in the last 12 months for at least one night, a zero if not.

Table 2 below shows that among those who reported being hospitalised in the post-baseline period, the most common cause of hospitalisation was ‘other’ or unspecified (43% of responses) followed by ‘pregnancy-related including delivery’ in 35% of responses followed by ‘injuries or accidents’ in 12% of responses. HIV-related care was the reason for hospitalisation for 6% of responses. The questionnaire did not probe individuals who answered that they were hospitalised for ‘other’ reasons.

**Table 2:** Reasons for reported hospitalisation, post-baseline survey rounds.

	All post-baseline survey rounds		HIV+		HIV-		South Africa		Zambia	
	Nr	%	Nr	%	Nr	%	Nr	%	Nr	%
HIV-related care including TB and other opportunistic infections	28	6.38	23	20.91	5	1.65	16	8.04	12	5.00
Pregnancy-related including delivery	153	34.85	29	26.36	118	38.94	73	36.68	80	33.33
Injuries or accidents	52	11.85	6	5.45	42	13.86	38	19.10	14	5.83
Other	190	43.28	48	43.64	128	42.24	63	31.66	127	52.92
No answer or missing variable	16	3.64	4	3.64	10	3.29	9	4.50	7	2.92
Total	439	100	110	100	303	100	199	100	240	100

Note: The breakdown by HIV status excludes 26 cases where the individual’s HIV status was unknown. Note that the HIV-related care categories includes TB and other opportunitistic infections and patients can have TB without having HIV, which plausibly explains why we see 5 HIV negative patient hospitalisations in this row.

S2 Table shows that hospitalisation was higher amongst PLWH and females, but there was no significant difference by country.

**Fig 1:**
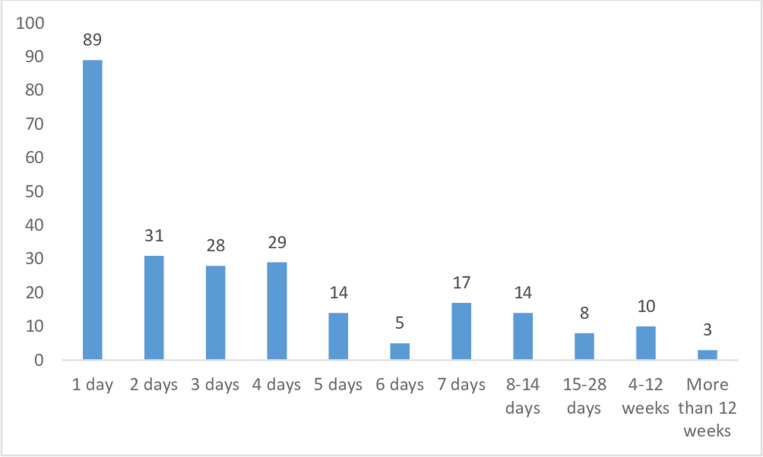
Bar graph for length of stay for most recent hospitalisation, pooled post-baseline subsample that reported hospitalisation (including hospital deliveries, injuries and accidents). Note: This information was captured in each survey and therefore can include more than one hospitalisation for the subsample of respondents who answered this module more than once.

Fig 1 below shows a bar graph for the number of days of hospital stay for the patient’s last hospitalisation in South Africa and Zambia. The most common duration of stay is 1 day, but there is a long tail to the right with three patients reporting hospitalisations exceeding twelve weeks (90 and 182 days).

Table 3 compares hospitalisation covariates across treatment and control arms at baseline. Outpatient or primary care utilisation did not differ across arms. Control sites had a slightly higher proportion of HIV-positive respondents, a greater proportion of respondents had completed secondary and tertiary education, and there were lower levels of severe poverty (quintile 1). At the baseline, hospitalisations in both arm A and arm B were lower than at the control sites in arm C, but this gap decreases when we exclude admissions for births, injuries, and accidents.

**Table 3:** Descriptive statistics: Covariates at baseline, per study arm.

	Arm A				Arm B				Arm C			
	Proportion	Lower bound 95% CI	Upper bound 95% CI	N	Proportion	Lower bound 95% CI	Upper bound 95% CI	N	Proportion	Lower bound 95% CI	Upper bound 95% CI	N
Outpatient care utilisation in last 3 months	0.0850	0.0727	0.0973	1977	0.0861	0.0744	0.0978	2218	0.1006	0.0882	0.1131	2236
HIV positive (lab result)	0.1990	0.1811	0.2168	1920	0.2085	0.1914	0.2256	2168	0.2246	0.2071	0.2422	2177
Male	0.2946	0.2746	0.3147	1989	0.2794	0.2608	0.2980	2237	0.2930	0.2742	0.3118	2249
Age 18–24	0.4017	0.3802	0.4233	1989	0.4005	0.3802	0.4209	2237	0.4046	0.3843	0.4249	2249
Age 25–34	0.3891	0.3677	0.4106	1989	0.3934	0.3731	0.4136	2237	0.3886	0.3685	0.4088	2249
Age 35+	0.2092	0.1913	0.2270	1989	0.2061	0.1893	0.2229	2237	0.2068	0.1900	0.2235	2249
Did not complete secondary education	0.6810	0.6604	0.7015	1981	0.6704	0.6509	0.6899	2230	0.6095	0.5893	0.6298	2241
Completed secondary education	0.3190	0.2985	0.3396	1981	0.3296	0.3101	0.3491	2230	0.3905	0.3702	0.4107	2241
Tertiary education	0.0671	0.0561	0.0782	1981	0.0502	0.0412	0.0593	2230	0.0710	0.0603	0.0816	2241
Poorest (Quintile 1)	0.2196	0.2007	0.2385	1844	0.2850	0.2656	0.3044	2081	0.1046	0.0918	0.1173	2219
Less poor (Quintile 2)	0.1871	0.1693	0.2049	1844	0.1994	0.1822	0.2166	2081	0.1803	0.1643	0.1963	2219
Middle (Quintile 3)	0.2082	0.1897	0.2268	1844	0.1898	0.1730	0.2067	2081	0.2379	0.2202	0.2557	2219
Less affluent (Quintile 4)	0.2223	0.2034	0.2413	1844	0.2009	0.1836	0.2181	2081	0.3064	0.2873	0.3256	2219
Most affluent quintile (Quintile 5)	0.1627	0.1458	0.1795	1844	0.1249	0.1107	0.1392	2081	0.1708	0.1551	0.1865	2219

Note: N reflects the number of responses to the specific hospitalisation question in the relevant subsample at PC0, with the proportions reflecting the share of the subsample who gave the specific response. The education categories do not add to 100% because all of those who obtained tertiary education also completed secondary education. CIs were adjusted for clustering associated with the study design.

The cluster-level analysis in Table 4 shows that there is no strong evidence that the intervention had any impact on hospitalisations excluding admissions for hospital delivery, accidents, and injuries. Among 30 alternative analyses, only one model specification finds evidence of a significant impact. We estimate a simple model without covariates, a pooled model with covariates as well as separate models for each of the three post-baseline surveys (PC12, PC24, and PC36). The primary analysis compares the hospitalisation risk in the two intervention arms (A + B) against the control arm C. However, given that Zambia and South Africa only introduced universal access to ART shortly before PC24 surveys started (see S1 Fig), we also investigate the impact of treatment arms A and B individually against the control arm. Comparisons may still be useful for PC24 because there were differences between ART eligibility between arm A and arms B and C for a large share of the preceding period. These differences were eliminated during the last 18 months of the intervention when universal ART became the standard of care. The estimates for the PC36 survey are included in this analysis of the individual treatment arms for the sake of completeness. All analyses are conducted for both the full sample and the PLWH subsample.

**Table 4:** Cluster-level analysis of impact of intervention on hospitalisations in the past 12 months, intervention arms (AB) vs. control arm (C).

	Without covariates, for all surveys excluding baseline	With covariates, for all surveys excluding baseline	With covariates, for survey 2 (PC12)	With covariates, for survey 3 (PC24)	With covariates, for end line survey (PC36)
**Primary analysis: Intervention arms vs. control arm (AB-C) for PLWH subsample**					
Adjusted risk ratio	0.9992 [0.52–1.92]	0.8231 [0.39–1.74]	0.7038 [0.34–1.44]	2.1417 [1.02–4.50]*	0.7858 [0.40–1.55]
Observations	21	21	21	21	21
**Primary analysis: Intervention arms vs. control arm (AB-C) for full sample**					
Adjusted risk ratio	0.9615 [0.57–1.61]	1.0288 [0.64–1.66]	0.8989 [0.43–1.87]	1.4611 [0.70–3.06]	0.9175 [0.44–1.89]
Observations	21	21	21	21	21
**Secondary analysis: Intervention arm vs. control arm (A-C) for PLWH subsample**					
Adjusted risk ratio	1.1031 [0.49–2.49]	0.7949 [0.20–3.23]	1.1962 [0.43–3.35]	2.6293 [0.84–8.24]	0.5417 [0.15–2.02]
Observations	14	14	14	14	14
**Secondary analysis: Intervention arm vs. control arm (A-C) for full sample**					
Adjusted risk ratio	1.022 [0.61–1.72]	1.0695 [0.59–1.95]	0.6081 [0.35–1.06]	1.8882 [0.59–6.08]	1.3101 [0.30–5.79]
Observations	14	14	14	14	14
**Secondary analysis: Intervention arm vs. control arm (B-C) for PLWH subsample**					
Adjusted risk ratio	0.9003 [0.42–1.88]	1.0184 [0.48–2.16]	0.8484 [0.34–2.14]	2.0040 [0.70–5.73]	0.7004 [0.50–0.97]
Observations	14	14	14	14	14
**Secondary analysis: Intervention arm vs. control arm (B-C) for full sample**					
Adjusted risk ratio	0.9037 [0.41–1.97]	1.11 [0.55–2.25]	1.3920 [0.45–4.35]	1.3001 [0.37–4.52]	0.5939 [0.24–1.46]
Observations	14	14	14	14	14

Note: In all cases, hospitalisations exclude hospitalisations due to admissions for birth, accidents and injuries. The survey asked individuals “In the last 12 months, how many times were you admitted to hospital or other types of inpatient care and stayed one or more nights?”. Our primary outcome variable is the resulting binary variable, which is 1 if the respondent had stayed in the hospital in the last 12 months for at least one night, a zero if not.” 95% CIs are reported in square brackets. * p < 0.05, **p < 0.01, ***p < 0.001. The results were estimated using Stata’s clan command. Control variables are baseline cluster-level HIV prevalence and cluster-level hospitalisations as well as gender, age categories, education categories, wealth index, and triplet categories. For the pooled analysis with all three post-baseline surveys, we also add a variable for the survey round. The risk ratio is calculated by generating R-values (the geometric mean of the observed cluster hospitalisation risks over the geometric mean of the expected cluster hospitalisation rates) for the intervention and control arms, and then dividing the intervention arm R-value by the control arm R-value.

## Discussion

Our study investigated how hospitalisation risks were impacted by community-wide delivery of the PopART combination prevention intervention. We found no significant relationship between study arms and the risk of hospitalisation – neither among the PLWH subsample nor the full sample. Comparing hospitalisations in the intervention and control arm clusters, the estimated adjusted risk ratio was 1.03 [0.64–1.66] for the full sample and 0.82 [0.39–1.74] for PLWH. Although contrary to our initial hypothesis, the absence of a significant impact is not unprecedented in the literature.

This evidence on hospitalisation is aligned with what is known about how the disease progresses and the reduction of risk of serious disease with early initiation [18,19]. A randomized controlled trial at 13 sites in 9 countries showed that HIV-1 disease progression was delayed and survival improved when ART was initiated early at CD4 counts of 350–550 cells/ µ L compared to late initiation (when CD4 count reaches 200–250 cells/ µ L, drops below 200 cells/ µ L, or when developing an AIDS-defining illness) [18]. The median increase in the CD4 count over the 2 years of study was 225 cells/ µ L for the early initiators and 37 cells/ µ L for late initiators. A study of a cohort of 3906 South African patients showed that both the risk of hospitalisation and the cost of hospitalisation were higher for PLWH with a CD4 cell count<100 cells/ µ L compared to PLWH with a CD4 cell count of 200–350 cells/ µ L [19].

A systematic review of the association between avoidable hospitalisation and primary health care access found 51 studies that met their eligibility criteria, the overwhelming majority (37) of which reported a significant inverse relationship between the two – 8 found no association and 5 found a positive relationship [20]. It was noted that the review was restricted to high-income countries. Similarly, an assessment of the literature examining the relationship between community health workers and healthcare utilisation for US patients with chronic diseases was equivocal, reporting that in 14 out of 34 eligible studies, healthcare supported by community health workers was associated with a statistically significant decline in hospitalisations and outpatient visits [21]. Of the seven RCTs that reported hospitalisations, six showed no significant decrease in hospitalisations relative to a control or a randomized observation group while one RCT observed a significant decrease.

A study of hospitalisations amongst a cohort of US PLWH attributed an observed decline in hospitalisations from 35 to 27 per 100 persons between 2002 and 2007, with a notable decrease in 2005, to improved availability of more convenient single-dose ART with fewer side effects [14]. The results from this study are not comparable to our study because it is an early-ART era observational study that relies on site-based routine data. Analysis from a rural surveillance site in South Africa showed significant increases in public sector primary health care visits, but significant declines in hospital visits for both HIV-positive and HIV-negative members of their population cohort during the rapid ART scale-up from 2009 to 2012 [22]. They conclude that their findings are consistent with a positive causal effect of improved access to ART, but acknowledge that their analysis does not allow them to establish causality.

A South African two-hospital study showed that hospitalisations were more likely at CD4 cell counts lower than 100 cells/ µ L compared to 200–350 cells/ µ L. These lower CD4 cell counts increased hospitalisations by 70% pre-ART and by 80% postART initiation [19]. Unfortunately, our study did not have data on CD4 cell counts to facilitate such analysis. An individually-randomised controlled study examining the impact of earlier initiation of treatment on a range of outcome variables (including hospitalisation) found there was no impact on unscheduled hospital admission – despite showing an impact on their composite primary outcome (serious AIDS-related, serious non–AIDS-related events or death from any cause). The study recruited 4685 patients from six geographic regions (Africa, Europe and Israel, North America, South America, Mexico, Australia, and Asia) and followed them for a mean of 3.0 years [12]. The immediate initiation group started treatment when their CD4 + count exceeded 500 cells/ µ L, while the delayed start group deferred treatment initiation until their CD4 + count had fallen to 350 cells/ µ L or at any point where the study participant developed AIDS.

We identified three hypothetical pathways for the impact of our study’s intervention on hospitalisations: (a) prevention of advanced HIV disease and associated conditions, (b) prevention of HIV infection in HIV-negative individuals, and (c) prevention of advanced TB disease. The latter pathway was expected to make a comparatively small contribution to hospitalisations due to the much lower prevalence of TB compared to HIV.

HIV prevention may only result in decreased hospitalisations over an extended period. The null result in a similar previous study with a 3-year time window suggests that this time frame may be too short to observe the impact on hospitalisation [12]. HIV disease progresses over several years: without treatment, the average life expectancy of PLWH is approximately 10 years [4,6].

This means that improved access to screening and testing via the CHiPs workers is the more likely pathway for an impact of the intervention on hospitalisation. Our HPTN071 study has shown a direct impact on HIV and TB testing, but such testing would only reduce hospitalisations if HIV and TB cases identified via CHiPs workers start treatment and adhere to treatment. Previous work on a different study in the same geographical areas as this study’s South African site has indicated that community-based testing had weak linkages to both HIV and TB care, with studies showing that 64% to 54% of patients testing positive at community sites start HIV treatment [23,24]. Reasons for not initiating ART after a positive HIV test included availability of time (61%), preferring to delay treatment initiation until feeling sick (48%), concerns about side effects (33%), and doubting the HIV diagnosis (16%) [25].

In the HPTN071 study, linkage to care was low in the first intervention rounds; with a median lag of 10 months for ART initiation after referral [26]. In the final intervention round, it was reported the median time from CHiP referral to ART initiation had declined to 3 months in both Zambia and South Africa and 70% of referrals reported ART initiation 12 months later [27]. Delayed linkage to care during the earlier intervention rounds may thus partly explain the null result for hospitalisation.

A further explanation for the null effect is that PC members (in all study arms) were offered a rapid on-the-spot HIV test at every survey, and would be assisted with linkage to care if found HIV-positive. This could dilute any effect of the intervention on rates of ART and viral suppression, and hence on hospitalisation. It is also possible that increased engagement with CHiPs and primary care services led to the detection of unrelated health conditions, potentially increasing hospital referrals and offsetting reductions in HIV-related hospitalisations.

### Strengths and limitations

This analysis was conducted within the structure of a large RCT with standardisation of interventions and enrolment and follow-up of the study cohort as well as extensive prospective work to ensure data accuracy. The hospitalisation questions were administered to a random subsample of the population, diminishing the sample size for this analysis, especially for HIV and TB-specific admissions. Our analysis was based on self-reported hospitalisations with a lack of detail on the reason for admissions. Our analysis is also limited by not having baseline CD4 cell count data. Participants were relatively young (between 18 and 47) and would have reported fewer hospitalisations than older populations. There was an overrepresentation of females in the study. Additionally, study power was limited due to the relatively low hospitalization rates and because this study was powered for HIV incidence as the primary outcome. The provision of on-the-spot HIV testing to PC participants in the control arm may have contributed to the null result, although the study team considered that it was important to offer this for ethical reasons. Furthermore, it is plausible that the study period may have been too short to fully capture the hospitalisation benefits of the intervention.

## Conclusions

Hospitalisations remain a major challenge for HIV high-burden settings, and putting pressure on medical resources and budgets. In this study, we investigated whether an intervention expanding access to screening for TB, testing for HIV and linkage to ART and adherence support had an impact on hospitalisation. Only one of 30 model specifications showed a significant impact of the intervention on hospitalisations, thus providing little evidence in support of the hypothesis that such interventions will reduce hospitalisations. The relatively low prevalence of TB (compared to HIV) may explain the lack of impact of TB screening on hospitalisations. The four-year time period of observation for this study was likely too short to capture the impact of the intervention on hospitalisation through preventing additional HIV cases. Take-up of ART following diagnosis improved over time, but the slow rates in early intervention rounds may have contributed to the null result.

Future studies seeking to answer this research question may benefit from a longer follow-up period. Achieving higher power would require a larger sample size or longer follow-up to accumulate more person-years of observation. Additionally, although not feasible for our study, measuring CD4 counts in all study arms could provide insights into the causal impact of increased treatment and viral suppression on hospitalisation.

## Supporting information

S1 Text Sample selection, study timelines and standard of care. (DOCX)

S1 Fig Timelines of HPTN 071 (PopART study) showing annual intervention rounds (R1-R3), Population cohort survey rounds (PC0-PC36), the primary analysis period and changes in ART initiation thresholds by country. *The dates shown for the start of universal ART refer to when this was implemented in study clinics in the respective countries. In Zambia, the first study clinic transitioned 19 April 2016 and the last 9 May 2016. This transition is represented by the dark purple band in the figure. In South Africa, the first study clinics transitioned on 10 October 2016 and the last on 21 November 2016. Source: HPTN 071 (PopART) Supplementary Material, Version 8.0 28 June 2019*.
(TIF)

S2 Text Compilation of the wealth index. (DOCX)

S2 Fig Enrollment, Follow-up and Hospitalisation of the Population Cohort. (TIF)

S1 Table Proportions of hospitalisations and hospitalisation covariates at baseline, by cluster Note: The education categories do not add to 100% because all of those who obtained tertiary education also completed secondary education. (DOCX)

S2 Table Proportions of hospitalisations by HIV status, country, gender and age [full sample] *Note: An adjusted Wald test was used to assess whether hospitalisation proportions differed significantly.* (DOCX)

S3 Table Cluster-level analysis of impact of intervention on hospitalisations (*including births, accidents and injuries*) in the past 12 months, intervention arms (AB) vs. control arm (C) *Notes: n all cases, hospitalisations exclude hospitalisations due to admissions for birth, accidents and injuries.* 95% CIs are reported in square brackets. * p < 0.05, **p < 0.01, ***p < 0.001.
(DOCX)
