# Supplementary material for: Does enhanced HIV prevention, diagnosis, and linkage to care reduce hospitalisation in high HIV-burden communities in Zambia and South Africa? findings from the HPTN 071 (PopART) randomised trial
Source: PLOS Glob Public Health. 2025 May 8;5(5):e0004373. doi: 10.1371/journal.pgph.0004373 (PMC12061103; doi:10.1371/journal.pgph.0004373)
Supplement: S1 Text — (DOCX) [file pgph.0004373.s001.docx]

**S1 Text. Sample selection, study timelines and standard of care**

The 21 study communities were initially organized into 7 matched triplets, with 4 in Zambia and 3 in South Africa, based on factors such as geographical proximity, HIV service partners, and the most reliable estimates of adult HIV prevalence. The matched design was chosen to reduce variability in baseline HIV incidence between communities, assuming it is linked to baseline HIV prevalence. While efforts were made to match based on geographical area, HIV service partners, and HIV prevalence, it was not feasible to match on the estimated ART uptake among HIV-positive individuals or the cluster size. This may slightly increase the variability in effect size across the triplets. However, using restricted randomisation will help maintain balance in average ART uptake and cluster size across the trial arms A, B, and C.

The 21 study communities of Zambia and South Africa had high HIV prevalence and incidence, and at the time were continuing to experience severe generalized HIV epidemics, with adult prevalence levels varying between a low of 11% and a high of 25% across the study communities. National estimates of HIV prevalence in adults aged 15 to 49 were 13.5% for Zambia and 17.8% for South Africa, and incidence estimates were 1.06% and 1.49%, respectively.^1^ Table 1 of the study protocol document lists for all 21 study communities the adult HIV prevalence and the percentage of PLWH on ART.^2^

The figure below is from the Supplementary Appendix of the paper on primary outcomes of the intervention and shows timelines for the study.^3^ The standard of care, most notably ART initiation thresholds, changed over the study periods. The dates shown for the start of universal ART (ART irrespective of a patient's CD4 cell count or WHO stage) refer to when this was implemented in study clinics in the respective countries. In Zambia, the first study clinic transitioned 19 April 2016 and the last 09 May 2016. This transition is represented by the dark purple band in the figure. In South Africa, the first study clinics transitioned 10 October 2016 and the last on 21 November 2016, a period represented by the dark purple band in the figure. Study personnel, clinic staff and implementing partners worked to ensure that study clinics implemented the new policy as soon as local guidelines and logistics such as staff training would allow. Transition to universal ART in study clinics, therefore, often preceded transition in neighbouring communities. This implies that over the course of the study the standard of care as it concerns criteria for ART initiation resembled more closely the intervention. The homebased testing and counselling, and other associated activities delivered by the CHIPs, remained as the difference between the intervention and arm C communities.

Protocol version 1.0 from 26 October 2012 specified that the study team would work with in-country health authorities to ensure to the degree possible that existing services in the seven control communities meet current local guidelines for HIV prevention and care. These activities include endeavoring to ensure that:

- Community members have adequate access to services for voluntary HIV counseling and testing.
- Referral services for male circumcision are available to men who are HIV uninfected and wish to be circumcised.
- HIV treatment and care are provided according to current local guidelines prior to version 3.0 of the protocol, and, upon implementation of version 3.0 of the protocol, will be provided to all HIV-infected clients attending an Arm C health center. The study team will endeavor to ensure that antiretroviral drugs are available to all patients who qualify for treatment, using the current ART drug regimen employed in the government program in each country.
- Adequate services for PMTCT are in place at antenatal and delivery services in the control communities.
- Treatment services for STIs and condoms are available through health units in accordance with local HIV prevention guidelines.

**References**

1. UNAIDS. Global report: UNAIDS report on the global AIDS epidemic. 2012. Geneva: UNAIDS; 2012.
2. Hayes, R., Ayles, H., Beyers, N. *et al.* HPTN 071 (PopART): Rationale and design of a cluster-randomised trial of the population impact of an HIV combination prevention intervention including universal testing and treatment – a study protocol for a cluster randomised trial. Trials. 2014;15, 57.

- Hayes RJ, Donnell D, Floyd S, et al. Effect of universal testing and treatment on HIV incidence — HPTN 071 (PopART). N Engl J Med. 2019; 381(3):207-218.
