# Supplementary figures and images for: Does enhanced HIV prevention, diagnosis, and linkage to care reduce hospitalisation in high HIV-burden communities in Zambia and South Africa? findings from the HPTN 071 (PopART) randomised trial
Source: PLOS Glob Public Health. 2025 May 8;5(5):e0004373. doi: 10.1371/journal.pgph.0004373 (PMC12061103; doi:10.1371/journal.pgph.0004373)

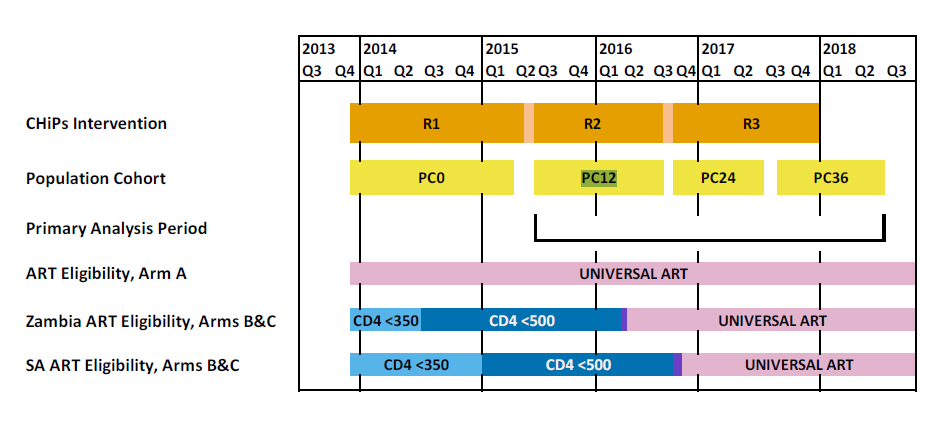

Supplement: S1 Fig — The dates shown for the start of universal ART refer to when this was implemented in study clinics in the respective countries. In Zambia, the first study clinic transitioned 19 April 2016 and the last 9 May 2016. This transition is represented by the dark purple band in the figure. In South Africa, the first study clinics transitioned on 10 October 2016 and the last on 21 November 2016. Source: HPTN 071 (PopART) Supplementary Material, Version 8.0 28 June 2019. (TIF) [file pgph.0004373.s002.tif]
