# Supplementary material for: Does enhanced HIV prevention, diagnosis, and linkage to care reduce hospitalisation in high HIV-burden communities in Zambia and South Africa? findings from the HPTN 071 (PopART) randomised trial
Source: PLOS Glob Public Health. 2025 May 8;5(5):e0004373. doi: 10.1371/journal.pgph.0004373 (PMC12061103; doi:10.1371/journal.pgph.0004373)
Supplement: S2 Text — (DOCX) [file pgph.0004373.s003.docx]

**S2 Text: Compilation of the wealth index**

This variable is created by running a principal component analysis (PCA) on a set of variables that are recoded in binary (0/1) format. The pool of assets included in the PCA estimation was access to a working cellphone, bicycle, car, electricity in house, TB, fridge/freezer, computer/laptop and a CD/MP3 player. The estimation also used variables that captured the household’s living conditions, including adequate sanitation (flush toilet or pit latrine), clean water (access to tap), borehole, electric cooker, flooring (parquet, lino, cement or tile), type of building structure. Due to lack of variation, some of the binary measures were excluded from the PCA, at a country-specific level. Zambia included all variables except improved drinking water source and improved cooking source; while South Africa included all 13 measures. Applying PCA separately to both countries, we used the first principal component to develop a standardized score.
