# Supplementary material for: Does enhanced HIV prevention, diagnosis, and linkage to care reduce hospitalisation in high HIV-burden communities in Zambia and South Africa? findings from the HPTN 071 (PopART) randomised trial
Source: PLOS Glob Public Health. 2025 May 8;5(5):e0004373. doi: 10.1371/journal.pgph.0004373 (PMC12061103; doi:10.1371/journal.pgph.0004373)

|  | **1** | **2** | **3** | **4** | **5** | **6** | **7** | **8** | **9** | **10** | **11** | **12** | **13** | **14** | **15** | **16** | **17** | **18** | **19** | **20** | **21** |
| --- | --- | --- | --- | --- | --- | --- | --- | --- | --- | --- | --- | --- | --- | --- | --- | --- | --- | --- | --- | --- | --- |
| Outpatient care utilisation in last 3 months | 0.27 | 0.15 | 0.13 | 0.05 | 0.04 | 0.07 | 0.20 | 0.15 | 0.15 | 0.23 | 0.06 | 0.18 | 0.08 | 0.05 | 0.10 | 0.01 | 0.02 | 0.05 | 0.07 | 0.02 | 0.06 |
| HIV positive  (lab result) | 0.13 | 0.15 | 0.23 | 0.19 | 0.15 | 0.26 | 0.23 | 0.18 | 0.20 | 0.27 | 0.25 | 0.22 | 0.27 | 0.27 | 0.28 | 0.27 | 0.35 | 0.17 | 0.11 | 0.10 | 0.02 |
| Male | 0.36 | 0.32 | 0.27 | 0.25 | 0.25 | 0.24 | 0.24 | 0.23 | 0.23 | 0.31 | 0.29 | 0.37 | 0.26 | 0.35 | 0.26 | 0.30 | 0.28 | 0.30 | 0.33 | 0.33 | 0.41 |
| Age 18-24 | 0.45 | 0.42 | 0.47 | 0.45 | 0.48 | 0.44 | 0.42 | 0.44 | 0.47 | 0.41 | 0.45 | 0.45 | 0.31 | 0.35 | 0.34 | 0.34 | 0.31 | 0.39 | 0.36 | 0.37 | 0.45 |
| Age 25-34 | 0.35 | 0.39 | 0.33 | 0.37 | 0.34 | 0.37 | 0.41 | 0.41 | 0.35 | 0.40 | 0.42 | 0.33 | 0.45 | 0.37 | 0.45 | 0.46 | 0.46 | 0.37 | 0.37 | 0.42 | 0.34 |
| Age 35 and more | 0.19 | 0.20 | 0.20 | 0.18 | 0.17 | 0.19 | 0.17 | 0.15 | 0.18 | 0.19 | 0.13 | 0.23 | 0.24 | 0.28 | 0.21 | 0.20 | 0.23 | 0.24 | 0.27 | 0.22 | 0.22 |
| Primary education | 0.47 | 0.38 | 0.49 | 0.66 | 0.92 | 0.87 | 0.72 | 0.81 | 0.69 | 0.60 | 0.66 | 0.62 | 0.60 | 0.65 | 0.58 | 0.58 | 0.54 | 0.66 | 0.65 | 0.56 | 0.71 |
| Completed secondary education | 0.53 | 0.62 | 0.51 | 0.34 | 0.08 | 0.13 | 0.28 | 0.19 | 0.31 | 0.40 | 0.34 | 0.38 | 0.40 | 0.35 | 0.42 | 0.42 | 0.46 | 0.34 | 0.35 | 0.44 | 0.29 |
| Tertiary education | 0.16 | 0.18 | 0.14 | 0.07 | 0.01 | 0.01 | 0.06 | 0.02 | 0.05 | 0.14 | 0.08 | 0.07 | 0.06 | 0.08 | 0.09 | 0.09 | 0.05 | 0.03 | 0.03 | 0.06 | 0.02 |
| Poorest (Quintile 1) | 0.05 | 0.03 | 0.03 | 0.03 | 0.45 | 0.70 | 0.14 | 0.23 | 0.13 | 0.24 | 0.20 | 0.23 | 0.35 | 0.36 | 0.11 | 0.07 | 0.18 | 0.15 | 0.11 | 0.07 | 0.04 |
| Less poor (Quintile 2) | 0.09 | 0.12 | 0.11 | 0.12 | 0.27 | 0.16 | 0.16 | 0.16 | 0.19 | 0.12 | 0.27 | 0.33 | 0.24 | 0.31 | 0.24 | 0.14 | 0.23 | 0.29 | 0.16 | 0.11 | 0.10 |
| Middle (Quintile 3) | 0.16 | 0.18 | 0.23 | 0.23 | 0.18 | 0.07 | 0.17 | 0.27 | 0.24 | 0.18 | 0.16 | 0.15 | 0.21 | 0.17 | 0.28 | 0.33 | 0.25 | 0.29 | 0.14 | 0.20 | 0.31 |
| Less affluent (Quintile 4) | 0.41 | 0.28 | 0.45 | 0.35 | 0.08 | 0.03 | 0.41 | 0.27 | 0.30 | 0.28 | 0.23 | 0.13 | 0.17 | 0.13 | 0.24 | 0.34 | 0.21 | 0.18 | 0.22 | 0.29 | 0.33 |
| Most affluent quintile (Quintile 5) | 0.29 | 0.40 | 0.18 | 0.27 | 0.02 | 0.04 | 0.12 | 0.07 | 0.14 | 0.19 | 0.15 | 0.16 | 0.02 | 0.03 | 0.13 | 0.12 | 0.13 | 0.10 | 0.37 | 0.33 | 0.22 |
| Any hospitalisation over the past 12 months | 0.06 | 0.04 | 0.06 | 0.03 | 0.03 | 0.03 | 0.04 | 0.04 | 0.06 | 0.04 | 0.04 | 0.03 | 0.03 | 0.04 | 0.11 | 0.02 | 0.05 | 0.03 | 0.11 | 0.05 | 0.04 |
| Any hospitalisation over the past 12 months excluding admissions for hospital delivery, injuries and accidents | 0.05 | 0.03 | 0.03 | 0.01 | 0.01 | 0.01 | 0.02 | 0.02 | 0.02 | 0.02 | 0.02 | 0.03 | 0.01 | 0.02 | 0.03 | 0.02 | 0.02 | 0.01 | 0.04 | 0.03 | 0.02 |

**Table S2: Proportions of hospitalisations by HIV status, country, gender and age [full sample]**

|  | **Any hospitalisation**  **over the past 12 months** | **Significance** | **Any hospitalisation over the past 12 months excluding admissions for hospital delivery, injuries, and accidents** | **Significance** |
| --- | --- | --- | --- | --- |
| HIV positive  (lab result) | 0.045 | 0.0075 | 0.029 | 0.0006 |
| HIV negative (lab result) | 0.033 |  | 0.015 |  |
| South Africa | .0402 | 0.2825 | 0.019 | 0.5008 |
| Zambia | .0326 |  | 0.017 |  |
| Male | 0.021 | 0.0000 | 0.014 | 0.0125 |
| Female | 0.042 |  | 0.020 |  |
| Age 18-24 | 0.365 | 0.8196 | 0.015 | 0.0561 |
| Age 25-34 | 0.038 | 0.3034 | 0.017 | 0.5957 |
| Age 35+ | 0.033 | 0.1619 | 0.023 | 0.0076 |

*Note: An adjusted Wald test was used to assess whether hospitalisation proportions differed significantly.*

**Table S3: Cluster-level analysis of impact of intervention on hospitalisations (*including births, accidents and injuries*) in the past 12 months, intervention arms (AB) vs. control arm (C)**

|  | **Without covariates, for all surveys excluding baseline** | **With covariates, for all surveys excluding baseline** | **With covariates, for survey 2 (PC12)** | **With covariates, for survey 3 (PC24)** | **With covariates, for end line survey (PC36)** |
| --- | --- | --- | --- | --- | --- |
| **Primary analysis: Intervention arms vs. control arm (AB-C) for PLWH subsample** | | | | | |
| Adjusted risk ratio | 0.8494 [0.49-1.48] | 0.7330 [0.38-1.43] | 0.8669 [0.43-1.76] | 1.1230 [0.59-2.16] | 1.1187 [0.46-2.70] |
| Observations | 21 | 21 | 21 | 21 | 21 |
| **Primary analysis: Intervention arms vs. control arm (AB-C) for full sample** | | | | | |
| Adjusted risk ratio | 0.8841 [0.55-1.42] | 0.9116 [0.59-1.42] | 0.8314 [0.39-1.80] | 1.1162 [0.57-2.20] | 0.9469 [0.45-2.00] |
| Observations | 21 | 21 | 21 | 21 | 21 |
| **Secondary analysis: Intervention arm vs. control arm (A-C) for PLWH subsample** | | | | | |
| Adjusted risk ratio | 0.8981 [0.47-1.70] | 0.6278 [0.18-2.19] | 0.7607 [0.22-2.67] | 1.7020 [0.80-3.62] | 0.5564 [0.15-2.00] |
| Observations | 14 | 14 | 14 | 14 | 14 |
| **Secondary analysis: Intervention arm vs. control arm (A-C) for full sample** | | | | | |
| Adjusted risk ratio | 0.9490 [0.50-1.82] | 0.8106 [0.46-1.42] | 0.4830 [0.23-1.00] | 1.2774 [0.52-3.15] | 0.9056 [0.25-3.28] |
| Observations | 14 | 14 | 14 | 14 | 14 |
| **Secondary analysis: Intervention arm vs. control arm (B-C) for PLWH subsample** | | | | | |
| Adjusted risk ratio | 0.8016 [0.40-1.63] | 0.8949 [0.48-1.68] | 1.2015 [0.60-2.42] | 1.2619 [0.65-2.44] | 0.8899 [0.62-1.27] |
| Observations | 14 | 14 | 14 | 14 | 14 |
| **Secondary analysis: Intervention arm vs. control arm (B-C) for full sample** | | | | | |
| Adjusted risk ratio | 0.8239 [0.41-1.97] | 1.0212 [0.61-1.70] | 1.2541 [0.76-2.06] | 1.0635 [0.29-3.90] | 0.9571 [0.41-2.25] |
| Observations | 14 | 14 | 14 | 14 | 14 |
| *In all cases, hospitalisations exclude hospitalisations due to admissions for birth, accidents and injuries. 95% CIs are reported in square brackets.*  ** p<0.05, **p<0.01, ***p<0.001.* | | | | | |
|  | | | | |  |

**List of legends**

**Fig. S1: Timelines of HPTN 071 (PopART study) showing annual intervention rounds (R1-R3), Population cohort survey rounds (PC0-PC36), the primary analysis period and changes in ART initiation thresholds by country**


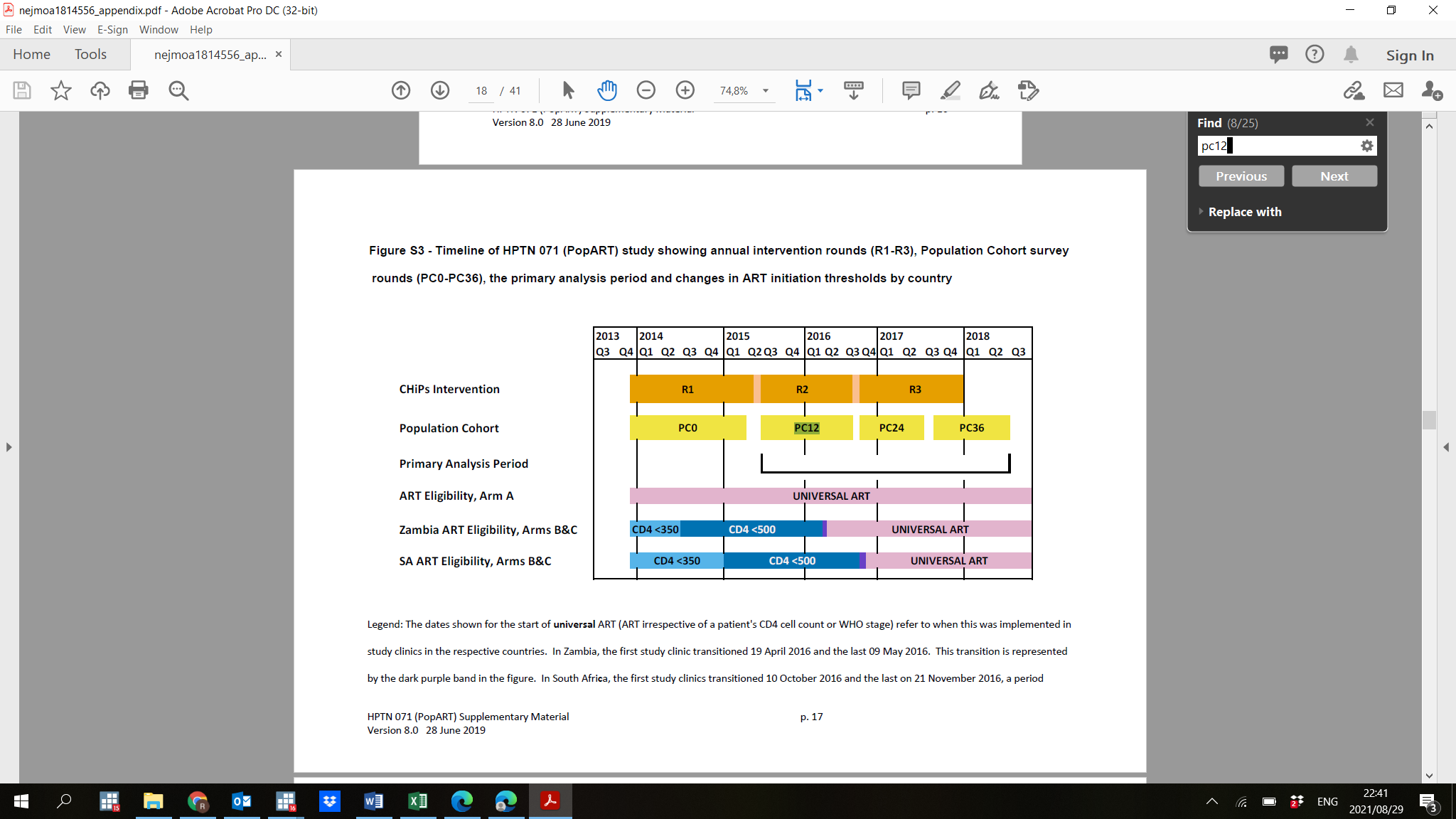


*Legend: The dates shown for the start of universal ART refer to when this was implemented in study clinics in the respective countries. In Zambia, the first study clinic transitioned 19 April 2016 and the last 9 May 2016. This transition is represented by the dark purple band in the figure. In South Africa, the first study clinics transitioned on 10 October 2016 and the last on 21 November 2016. Source: HPTN 071 (PopART) Supplementary Material, Version 8.0 28 June 2019*

**Fig. S2: Enrollment, Follow-up and Hospitalisation of the Population Cohort**


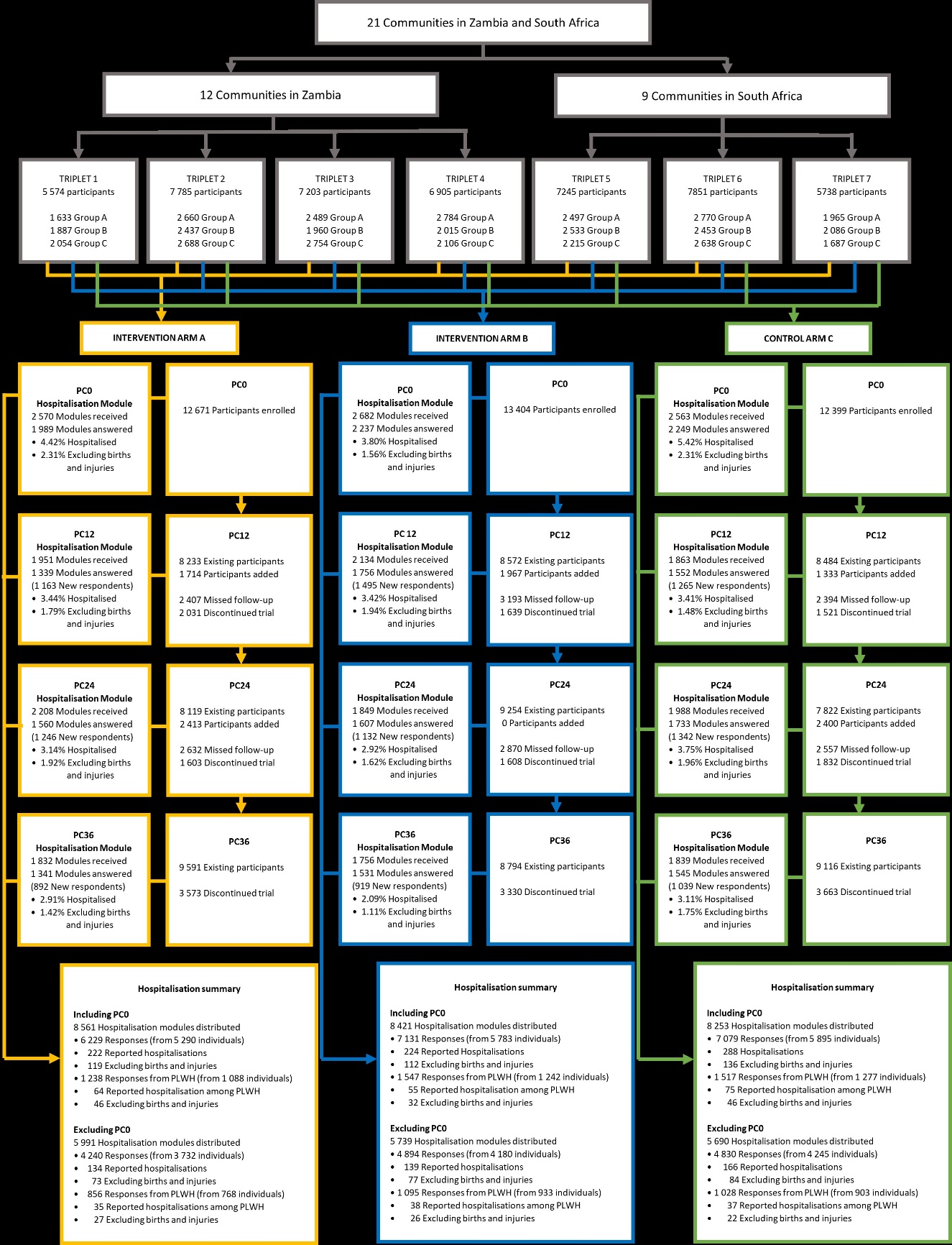

Supplement: S1 Table — (DOCX) [file pgph.0004373.s005.docx]
