# Supplementary material for: Does enhanced HIV prevention, diagnosis, and linkage to care reduce hospitalisation in high HIV-burden communities in Zambia and South Africa? findings from the HPTN 071 (PopART) randomised trial
Source: PLOS Glob Public Health. 2025 May 8;5(5):e0004373. doi: 10.1371/journal.pgph.0004373 (PMC12061103; doi:10.1371/journal.pgph.0004373)
Supplement: S2 Table — (DOCX) [file pgph.0004373.s006.docx]

|  | **Any hospitalisation**  **over the past 12 months** | **Significance** | **Any hospitalisation over the past 12 months excluding admissions for hospital delivery, injuries, and accidents** | **Significance** |
| --- | --- | --- | --- | --- |
| HIV positive  (lab result) | 0.045 | 0.0075 | 0.029 | 0.0006 |
| HIV negative (lab result) | 0.033 |  | 0.015 |  |
| South Africa | .0402 | 0.2825 | 0.019 | 0.5008 |
| Zambia | .0326 |  | 0.017 |  |
| Male | 0.021 | 0.0000 | 0.014 | 0.0125 |
| Female | 0.042 |  | 0.020 |  |
| Age 18-24 | 0.365 | 0.8196 | 0.015 | 0.0561 |
| Age 25-34 | 0.038 | 0.3034 | 0.017 | 0.5957 |
| Age 35+ | 0.033 | 0.1619 | 0.023 | 0.0076 |
