# Supplementary material for: Does enhanced HIV prevention, diagnosis, and linkage to care reduce hospitalisation in high HIV-burden communities in Zambia and South Africa? findings from the HPTN 071 (PopART) randomised trial
Source: PLOS Glob Public Health. 2025 May 8;5(5):e0004373. doi: 10.1371/journal.pgph.0004373 (PMC12061103; doi:10.1371/journal.pgph.0004373)
Supplement: S3 Table — 95% CIs are reported in square brackets. * p < 0.05, **p < 0.01, ***p < 0.001. (DOCX) [file pgph.0004373.s007.docx]

|  | **Without covariates, for all surveys excluding baseline** | **With covariates, for all surveys excluding baseline** | **With covariates, for survey 2 (PC12)** | **With covariates, for survey 3 (PC24)** | **With covariates, for end line survey (PC36)** |
| --- | --- | --- | --- | --- | --- |
| **Primary analysis: Intervention arms vs. control arm (AB-C) for PLWH subsample** | | | | | |
| Adjusted risk ratio | 0.8494 [0.49-1.48] | 0.7330 [0.38-1.43] | 0.8669 [0.43-1.76] | 1.1230 [0.59-2.16] | 1.1187 [0.46-2.70] |
| Observations | 21 | 21 | 21 | 21 | 21 |
| **Primary analysis: Intervention arms vs. control arm (AB-C) for full sample** | | | | | |
| Adjusted risk ratio | 0.8841 [0.55-1.42] | 0.9116 [0.59-1.42] | 0.8314 [0.39-1.80] | 1.1162 [0.57-2.20] | 0.9469 [0.45-2.00] |
| Observations | 21 | 21 | 21 | 21 | 21 |
| **Secondary analysis: Intervention arm vs. control arm (A-C) for PLWH subsample** | | | | | |
| Adjusted risk ratio | 0.8981 [0.47-1.70] | 0.6278 [0.18-2.19] | 0.7607 [0.22-2.67] | 1.7020 [0.80-3.62] | 0.5564 [0.15-2.00] |
| Observations | 14 | 14 | 14 | 14 | 14 |
| **Secondary analysis: Intervention arm vs. control arm (A-C) for full sample** | | | | | |
| Adjusted risk ratio | 0.9490 [0.50-1.82] | 0.8106 [0.46-1.42] | 0.4830 [0.23-1.00] | 1.2774 [0.52-3.15] | 0.9056 [0.25-3.28] |
| Observations | 14 | 14 | 14 | 14 | 14 |
| **Secondary analysis: Intervention arm vs. control arm (B-C) for PLWH subsample** | | | | | |
| Adjusted risk ratio | 0.8016 [0.40-1.63] | 0.8949 [0.48-1.68] | 1.2015 [0.60-2.42] | 1.2619 [0.65-2.44] | 0.8899 [0.62-1.27] |
| Observations | 14 | 14 | 14 | 14 | 14 |
| **Secondary analysis: Intervention arm vs. control arm (B-C) for full sample** | | | | | |
| Adjusted risk ratio | 0.8239 [0.41-1.97] | 1.0212 [0.61-1.70] | 1.2541 [0.76-2.06] | 1.0635 [0.29-3.90] | 0.9571 [0.41-2.25] |
| Observations | 14 | 14 | 14 | 14 | 14 |
|  | | | | | |
